# Association of antipsychotic use with breast cancer: a systematic review and meta-analysis of observational studies with over 2 million individuals

**DOI:** 10.1017/S2045796022000476

**Published:** 2022-09-05

**Authors:** Janice Ching Nam Leung, Dora Wai Yee Ng, Rachel Yui Ki Chu, Edward Wai Wa Chan, Lei Huang, Dawn Hei Lum, Esther Wai Yin Chan, Daniel J. Smith, Ian Chi Kei Wong, Francisco Tsz Tsun Lai

**Affiliations:** 1 Centre for Safe Medication Practice and Research, Department of Pharmacology and Pharmacy, Li Ka Shing Faculty of Medicine, The University of Hong Kong, Hong Kong SAR, People's Republic of China; 2 Laboratory of Data Discovery for Health (D24H), Hong Kong Science Park, Hong Kong Science and Technology Park, Hong Kong SAR, People's Republic of China; 3 Centre for Clinical Brain Sciences, Division of Psychiatry, College of Medicine & Veterinary Medicine, The University of Edinburgh, Edinburgh, Scotland, UK; 4 Research Department of Practice and Policy, School of Pharmacy, University College London, London, UK; 5 Aston School of Pharmacy, Aston University, Birmingham, UK

**Keywords:** Antipsychotics, chronic conditions, drug side effects other, epidemiology, multimorbidity

## Abstract

**Aims:**

Despite reports of an elevated risk of breast cancer associated with antipsychotic use in women, existing evidence remains inconclusive. We aimed to examine existing observational data in the literature and determine this hypothesised association.

**Methods:**

We searched Embase, PubMed and Web of Science™ databases on 27 January 2022 for articles reporting relevant cohort or case-control studies published since inception, supplemented with hand searches of the reference lists of the included articles. Quality of studies was assessed using the Newcastle-Ottawa Scale. We generated the pooled odds ratio (OR) and pooled hazard ratio (HR) using a random-effects model to quantify the association. This study was registered with PROSPERO (CRD42022307913).

**Results:**

Nine observational studies, including five cohort and four case-control studies, were eventually included for review (*N* = 2 031 380) and seven for meta-analysis (*N* = 1 557 013). All included studies were rated as high-quality (seven to nine stars). Six studies reported a significant association of antipsychotic use with breast cancer, and a stronger association was reported when a greater extent of antipsychotic use, e.g. longer duration, was operationalised as the exposure. Pooled estimates of HRs extracted from cohort studies and ORs from case-control studies were 1.39 [95% confidence interval (CI) 1.11–1.73] and 1.37 (95% CI 0.90–2.09), suggesting a moderate association of antipsychotic use with breast cancer.

**Conclusions:**

Antipsychotic use is moderately associated with breast cancer, possibly mediated by prolactin-elevating properties of certain medications. This risk should be weighed against the potential treatment effects for a balanced prescription decision.

## Introduction

Antipsychotic medications are widely prescribed for people living with mental disorders such as schizophrenia, bipolar disorder, major depressive disorder and dementia, with an increasing trend of off-label use also observed worldwide in recent decades (Hálfdánarson *et al.*, 2017; Ng *et al.*, 2021). Despite a more tolerable safety profile of second-generation antipsychotic medications (Herrmann *et al.*, 2004), metabolic and endocrinologic abnormalities associated with antipsychotic use have been observed (De Hert *et al.*, 2012). These abnormalities may represent pathomechanisms underlying the known association of antipsychotic use with a range of relatively rare adverse events such as stroke and myocardial infarction (Douglas and Smeeth, 2008; Lai *et al.*, 2020).

Some studies have also reported an elevated cancer incidence related to the use of antipsychotics (Dalton *et al.*, 2006; Nielsen *et al.*, 2017). It has been shown women living with schizophrenia and bipolar disorder have a higher risk of developing breast cancer compared with the general population (Chou *et al.*, 2017; Anmella *et al.*, 2021) and antipsychotic use may potentially explain at least part of this increased risk. This is supported by a widely adopted working hypothesis of the hyperprolactinaemia-inducing property of certain antipsychotics such as pimozide, risperidone and clomipramine (De Hert *et al.*, 2016*b*; Johnston *et al.*, 2018). Other possible mechanisms may include poorer lifestyles regarding self-care and health consciousness among antipsychotic users (Bly *et al.*, 2014), as well as the commonly reported antipsychotic-mediated weight gain (Balt *et al.*, 2011). With complex mechanisms and likely multiple interacting risk factors, existing evidence remains inconclusive, and no definitive conclusion could be drawn regarding this association. Furthermore, although safety monitoring is an integral component of randomised controlled trials, the study design's inherent weaknesses such as insufficient sample size for rare outcomes, discrepancies in adverse event reporting and inadequate follow-up period to capture cancer incidence (Hughes *et al.*, 2014; Phillips *et al.*, 2019) pose as a challenge to investigate this association. Longitudinal observational data are therefore considered much more suitable for this enquiry.

A synthesis of the existing published data is important to inform clinical practices with regards to the prescription of antipsychotic medications in consideration of the potentially elevated risk of breast cancer. This synthesis will inform the risk–benefit assessment of antipsychotic use in facilitation of an optimal prescription decision and treatment outcome. In this study, we aim to systematically review and conduct a meta-analysis on the existing evidence to determine the association of antipsychotic use with breast cancer.

## Methods

### Search strategy and eligibility

We followed the Preferred Reporting Items for Systematic Reviews and Meta-Analyses (PRISMA) checklist in conducting this review (Page *et al.*, 2021). As this meta-analysis was based on published data, ethics approval was not required. In accordance with a protocol registered with PROSPERO (Ref: CRD42022307913), we performed preliminary scoping searches to identify databases with substantial pharmacoepidemiologic evidence on the topic. Based on the results of our preliminary searches, we conducted a systematic search of articles published in English in peer-reviewed scholarly journals in respective electronic databases, namely PubMed, Embase and Web of Science™ from inception. The last search was conducted on 27 January 2022. The search strategy was developed based on two subjects: antipsychotics and breast cancer. Search terms and combinations of Medical Subject Headings (MeSH), keywords and text words were derived from previously published systematic reviews (Moja *et al.*, 2012; Indave *et al.*, 2016; Krause *et al.*, 2018) on the two subjects and were selected for each database to optimise sensitivity and specificity of the search. Hand searches through the reference lists of included articles were conducted to avoid the omission of relevant research. For details of specific search keywords and strategies, refer to online Supplementary eTable 1.

All published cohort and case-control observational studies that investigated and quantified the association of antipsychotic use (*v*. non-use) with breast cancer in individuals aged 16 or above were considered for inclusion in the review. Studies were excluded if they were not published in English, had a study design that was neither cohort nor case-control, included participants who developed breast cancer prior to antipsychotic exposure or did not compare antipsychotic use to non-use, such as comparing between different classes of antipsychotics.

### Extraction

Study eligibility was independently determined by JCNL and DWYN. Cohen's kappa was computed to indicate interrater reliability. Data extraction was completed simultaneously using a standardised data extraction form. Data regarding the context, population, intervention, outcome and measures of association of each study were extracted and recorded in the form. Discrepancies were reconciled through discussion and consultation with a senior author (FTTL).

### Quality assessment of included studies

The methodological quality of each included study was assessed using the Newcastle-Ottawa Scale (NOS). Like the data extraction procedure, the quality assessment was conducted independently by JCNL and DWYN. Study quality was indicated by numbers of stars, with nine representing the highest possible methodological rigour. See online Supplementary eTable 3 for details of the quality assessment procedures. Cohen's kappa was not calculated for the quality assessment decisions, as nine studies were included and there were only a few discrepancies, which were resolved through in-depth discussions.

### Pooled estimates

Upon satisfactory assessment result with regards to multivariable adjustment according to the NOS, meta-analyses of the estimates of the association, i.e. odds ratios (ORs) and hazard ratios (HRs), were conducted. Stratified by study design, i.e. cohort and case-control studies, the estimates of the association of antipsychotic use and breast cancer were pooled using a random effects model. The exposure was binarily operationalised as any antipsychotic use compared with non-use. In cases where this operationalisation was not possible, the longest-term exposure category, or the category representing the farthest extent of antipsychotic use, were used in comparison with non-use in the pooled estimates. The inverse variance weighting method was used to determine the relative importance between studies while the *I*^2^ statistic was used to examine the heterogeneity of the estimates across studies. Upon a sufficient number of included studies, the Egger's regression test was conducted to detect any publication bias in the pooled estimates. The pooled estimates and test for heterogeneity were implemented using Cochrane Collaboration Review Manager (Version 5.4.1).

## Results

**Fig. 1.**
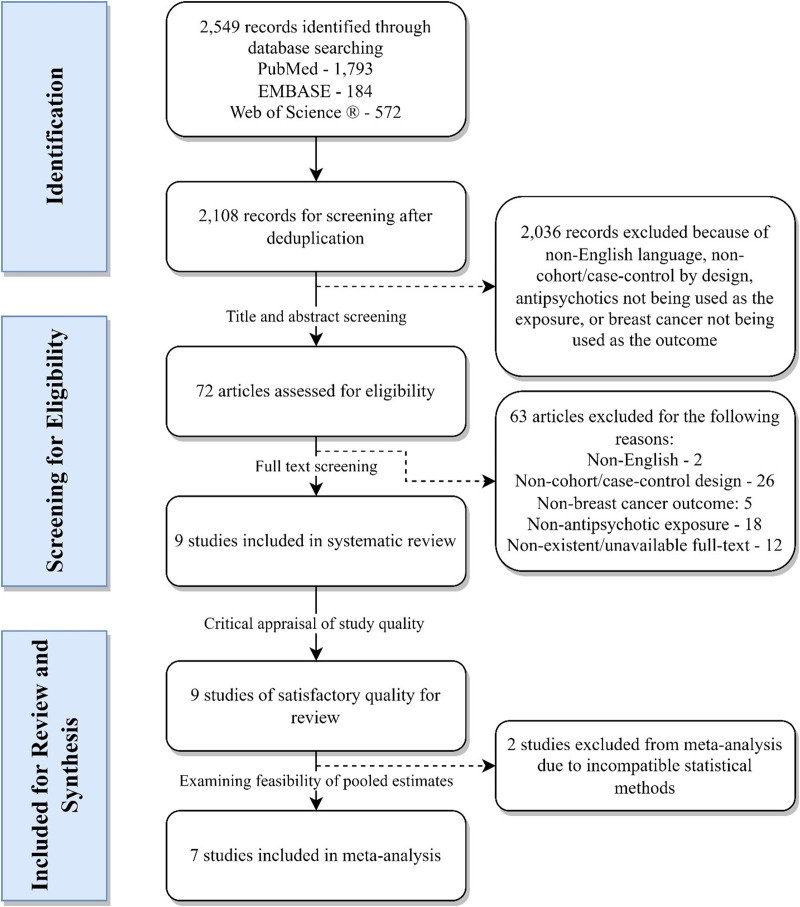
Flow chart of article selection.

As shown in Fig. 1, upon initial search, we retrieved a total of 2549 articles from electronic databases, of which 441 were removed as duplicates. The title and abstract screening process further excluded 2036 articles published in non-English languages, using a study design other than cohort or case-control, not adopting breast cancer as the outcome or not using antipsychotic use as the exposure. After carefully examining the eligibility of the remaining 72 articles by full-text, nine studies (*N* =  2 031 380) were included for a qualitative synthesis and quality assessment. Cohen's kappa for title and abstract screening [0.496, 95% confidence interval (CI) 0.404–0.588] and full-text selection (0.742, 95% CI 0.567–0.917) suggest moderate and substantial agreement respectively. Two studies were excluded from the meta-analysis (Mortensen, 1987; Dalton *et al.*, 2006), as the effect measures summarising the association were incomparable to that of the other studies and the use of incompatible statistical methods. Seven included studies (*N* = 1 557 013) provided adequate data for a pooled estimate of the hypothesised association. Study characteristics and results, as well as quality assessment scores are tabulated in Tables 1 and 2.

**Table 1.** Characteristics and results of the critical appraisal of included studies (*N* = 9)

Study	Data source	Study period	Region	Study design	Inclusion criteria	Exclusion criteria (no need diagnosis codes)	Outcome definition	Scores from Newcastle Ottawa Scale		
								Selection	Comparability	Exposure/outcome
Chou *et al*. (2017)	LHID2000; RCIPD	1998–2011	Taiwan	C	Exposed: female schizophrenia patients with AP prescription between 1998 and 2008; non-exposed: females without mental illness and no AP prescription	Diagnosed with BC before or within 1 year after the schizophrenia diagnosis	BC diagnosis (ICD-9-CM)	****	**	**
Dalton *et al*. (2006)	CPR; Danish Cancer Registry; North Jutland Prescription Database	01/01/1989–31/12/2002	Denmark	C	Danish women aged 16–85 years of age	History of cancer diagnosis before 1989 or age of 16 years	First primary diagnosis of cancer	****	**	***
George *et al*. (2020)	WHI	1993–31/03/2018	United States	C	Postmenopausal women aged 50–79 years	History of BC; <1 day follow-up time	BC diagnosis	***	**	**
Hippisley-Cox *et al*. (2007)	QRESEARCH	01/01/1995–01/07/2005	United Kingdom	CC	Patients aged 25–100 years; had ⩾12 months computerised medical record data before index date	History of cancer diagnosis before index date; BC cases or controls with mastectomy/tamoxifen use record ⩾12 months before first record of BC	First-ever record of index cancer including post-mortem diagnosis	****	**	**
Mortensen (1987)	Census population; Danish Cancer Registry	1957–1980	Denmark	CC	Schizophrenia inpatients in Danish psychiatric hospitals on 26/09/1957	–	BC diagnosis	**	**	***
Pottegård *et al*. (2018)	Danish Cancer Registry; Danish National Prescription registry; Danish National Patient Register; Danish Pathology Registry; Danish Psychiatric Central Register; Statistics Denmark; Danish Civil Registration System	1995–2015	Denmark	CC	Women with first-time diagnosis of invasive breast cancer during study period; had ⩾5 years of prescription data	Women outside age range of 18–85 years at index date; resided outside of Denmark within 10 years prior to index date; history of cancer or mastectomy	Histologically verified BC diagnosis (ICD-10)	****	**	**
Rahman *et al*. (2022)	IBM Marketscan Commercial; Multi-State Medicaid Databases	01/01/2007–30/06/2016	United States	C	Women aged 18–64; patients with records of claims from insurance programme for at least 12 months of before prescription of antipsychotic, anticonvulsant or lithium	Women with exposure to prochlorperazine only; patients with prescription drug claim for tamoxifen, a diagnosis of BC without treatment or any history of BC before index date; the first fill of antipsychotics did not fall within a continuous enrolment period	BC diagnosis (ICD-9/10) with pathologic verification, or BC diagnosis with evidence of surgical treatment or chemotherapy	****	**	**
Taipale *et al*. (2021)	Finnish hospital discharge register, prescription register, cancer register	1995–31/12/2017	Finland	CC	Women aged ⩾16 years; had diagnosis of schizophrenia between 1972 and 2014	History of cancer diagnosis (except for non-melanoma skin cancer), receipt of organ transplant, mastectomy or diagnosis of HIV	First invasive BC diagnosis (ICD-10) between 2000 and 2017, with histological verification at age between 18 and 85 years	****	**	***
Wang *et al*. (2002)	NJ Medicaid; PAAD, NJ Medicare, NJ Cancer Registry	01/01/1989–30/06/1995	New Jersey, United States	C	Women aged ⩾20 years; had ⩾1 medical service/prescription in each of 2 consecutive 6-month periods	Non-exposed subject with previously/subsequently filled AP prescription; BC diagnosis, BC surgical procedure or related hospitalisation or tamoxifen citrate prescription on or 3 months after 1 year of enrolment in a benefits programme (Medicaid/PAAD)	First BC diagnosis (ICD-Oncology V2) at least 3 months after index date or had first claim for BC surgery or hospitalisation for BC surgery	****	**	***

C, cohort; CC, case-control; GPRD, General Practice Research Database; LHID2000, Longitudinal Health Insurance Database 2000; RCIPD, Registry for Catastrophic Illness Patient Database; CPR, Central Population Register; WHI, Women's Health Initiative cohort; NJ Medicaid, New Jersey Medicaid; PAAD, New Jersey Pharmaceutical Assistance to the Aged and Disabled; NJ Medicare, New Jersey Medicare; NJ Cancer Registry, New Jersey Cancer Registry; AP, antipsychotics; BC, breast cancer; ICD, International Classification of Diseases.

**Table 2.** Results of included studies (*N* = 9)

Study	Sample size	Exposed group definition	Number of cases in ‘exposed group’	Association of antipsychotic use with breast cancer (BC)	Adjusted covariates
Chou *et al*. (2017)	Exposed: 10 727 Non-exposed: 10 727	Had FGA, SGA or both FGA and SGA prescription	119	HR: 1.94 (1.43–2.63)	Age, occupation, monthly income, comorbidities, medication (lithium, valproate sodium, antidepressants, anxiolytics and hypnotics)
Dalton *et al*. (2006)	Exposed: 25 264 Non-exposed: 448 983	⩾2 neuroleptic medication prescription (ATC: N05A)	258	IRR: 1.06 (0.93–1.21)	Age, hospitalisations for COPD, liver cirrhosis/alcoholism, ever use of NSAID/HT, number of children, age at first birth
George *et al*. (2020)	Exposed: 642 Non-exposed: 155 095	Self-reported AP medication (UpToDate)	Invasive BC: Typical AP: 10 Atypical AP: 4 *In situ* BC: Typical AP: 7	Invasive BC: Typical AP HR: 0.67 (0.36–1.25) Atypical AP HR: 1.45 (0.54–3.87) *In situ* BC: Typical AP HR: 2.05 (0.97–4.30)	Age, WHI participation, HT trial arm
Hippisley-Cox *et al*. (2007)	BC cases: 10 535 BC controls: 50 074	⩾1 prescription of AP (conventional, atypical, lithium)	40	OR: 1.55 (1.08–2.23)	Age, obesity, use of oral contraceptives/HT, smoking, BMI, Townsend score, comorbidities, medications, other serious mental health conditions
Mortensen (1987)	BC cases: 40 BC controls: 80	Exposure expressed as mean yearly number of defined daily doses (1 DDD = 300 mg chlorpromazine)	40	Haloperidol user cancer incidence ratio: 0.3 (*p* = 0.03) Neuroleptics (excluding reserpine and haloperidol) cancer incidence ratio: 0.4 (*p* = 0.09)	Age at first admission, length of stay in psychiatric hospital, ECT, other shock treatment, lobotomy, neuroleptic treatment, no. chest X-rays, social group, marital status, residence, occupation, childbirths, alcohol/drug abuse
Pottegård *et al*. (2018)	Cases: 60 360 Controls: 603 600	Cumulative exposure since 1995 until 1 year prior index date	Ever use: 4798 Long-term use: 693	AP ever use OR: 1.00 (0.97–1.04) Prolactin-inducing AP long-term use OR: 1.18 (1.06–1.32)	Age, use of drugs known/suspected to modify BC risk, prior diagnoses of diabetes, COPD and alcohol-related disease, prior psychiatric diagnoses, Charlson comorbidity index scores, highest achieved education
Rahman *et al*. (2022)	Exposed: 312 702 Non-exposed: 228 035	Had outpatient prescription drug claim with at least 1 day's supply for antipsychotics	914	HR: 1.35 (1.14–1.61)	Age, HT, diabetes, obesity, alcohol abuse, pre-existing benign breast disease, Medicaid enrolment, mental health diagnoses
Taipale *et al*. (2021)	Cases: 1069 Controls: 5339	Had antipsychotic prescription until 1 year before cancer diagnosis	Exposed 1–4 years: 108 Exposed ⩾5 years: 830	Exposed 1–4 years OR 1.18 (0.86–1.62) Exposed ⩾5 years: OR 1.74 (1.38–2.21)	Age, diagnoses of CVD/diabetes/asthma/COPD, substance misuse, suicide attempt, number of children, use and duration of use of drugs potentially modifying risk of BC
Wang *et al*. (2002)	Exposed: 52 819 Non-exposed: 55 289	AP prescription 1 year before index date; had 2 other AP prescriptions not used for psychiatric indications	1239	HR: 1.16 (1.07–1.26)	Age, race, socioeconomic status, benign breast disorders, obesity, non-breast malignancies, Charlson comorbidity score, no. medical outpatient visits, nursing home use

FGA, first-generation antipsychotics; SGA, second-generation antipsychotics; ATC N05A, Anatomical Therapeutic Chemical Classification System (Antipsychotics); AP, antipsychotics; BC, breast cancer; HT, hormone replacement therapy; NSAID, nonsteroidal anti-inflammatory drugs; COPD, chronic obstructive pulmonary disease; WHI, Women's Health Initiative cohort; ECT, electroconvulsive therapy.

### Study characteristics

The included studies have been conducted in five countries/jurisdictions: three studies in the United States (Wang *et al.*, 2002; George *et al.*, 2020; Rahman *et al.*, 2022), three studies in Denmark (Mortensen, 1987; Dalton *et al.*, 2006; Pottegård *et al.*, 2018) and one study each in Finland (Taipale *et al.*, 2021), Taiwan (Chou *et al.*, 2017) and the United Kingdom (Hippisley-Cox *et al.*, 2007). Of the nine studies, five were cohort studies (Wang *et al.*, 2002; Dalton *et al.*, 2006; Chou *et al.*, 2017; George *et al.*, 2020; Rahman *et al.*, 2022) and four were case-control studies (Mortensen, 1987; Hippisley-Cox *et al.*, 2007; Pottegård *et al.*, 2018; Taipale *et al.*, 2021). The study sample sizes range from 120 (Mortensen, 1987) to over 0.6 million (Pottegård *et al.*, 2018) individuals. All studies received a moderate to high score in the quality assessment ranging from seven to nine stars based on the criteria of NOS. Six studies (Wang *et al.*, 2002; Hippisley-Cox *et al.*, 2007; Chou *et al.*, 2017; Pottegård *et al.*, 2018; Taipale *et al.*, 2021; Rahman *et al.*, 2022) reported a significant association between antipsychotic use (various operationalisations) and breast cancer development.

### Outcome – breast cancer

All nine studies defined the outcome of interest as the first-time diagnosis of breast cancer, with five studies specifying the adopted diagnosis explicitly based on International Classification of Diseases (ICD) (Wang *et al.*, 2002; Chou *et al.*, 2017; Pottegård *et al.*, 2018; Taipale *et al.*, 2021; Rahman *et al.*, 2022), one of which also identified first claims of breast cancer surgeries without an ICD code diagnosis as cases (Wang *et al.*, 2002). Either surgery, chemotherapy or hospitalisation for breast cancer in addition to the diagnosis with ICD code was adopted for one study (Rahman *et al.*, 2022). Three studies (Pottegård *et al.*, 2018; Taipale *et al.*, 2021; Rahman *et al.*, 2022) used a histological or pathologic verification for the breast cancer diagnosis. Post-mortem diagnosis of breast cancer in cases who died was also used to define cases in a case-control study (Hippisley-Cox *et al.*, 2007).

Three studies (Pottegård *et al.*, 2018; Taipale *et al.*, 2021; Rahman *et al.*, 2022) that included additional verification like histology received at least eight out of nine stars in the quality assessment. All three studies (Pottegård *et al.*, 2018; Taipale *et al.*, 2021; Rahman *et al.*, 2022) reported a significant association. All five studies (Wang *et al.*, 2002; Chou *et al.*, 2017; Pottegård *et al.*, 2018; Taipale *et al.*, 2021; Rahman *et al.*, 2022) that specified the diagnosis based on ICD codes supported the association. From the remaining studies that received quality assessment scores ranging from seven to nine stars (Mortensen, 1987; Dalton *et al.*, 2006; Hippisley-Cox *et al.*, 2007; Chou *et al.*, 2017; George *et al.*, 2020), both association and non-association were observed.

### Confounder adjustment

Confounder adjustment applied in nine studies can be summarised into three main categories, namely clinical history; lifestyle and socioeconomic factors. All nine studies adjusted for covariates related to age and clinical history. In particular, the use of drugs known or suspected to modify breast cancer risk such as lithium, oral contraceptives or hormone replacement therapy were adjusted in seven out of nine studies (Dalton *et al.*, 2006; Hippisley-Cox *et al.*, 2007; Chou *et al.*, 2017; Pottegård *et al.*, 2018; George *et al.*, 2020; Taipale *et al.*, 2021; Rahman *et al.*, 2022).

Adjusted lifestyle factors include obesity, smoking, body mass index (BMI) and substance misuse. Five of the nine studies had made such adjustments (Mortensen, 1987; Wang *et al.*, 2002; Hippisley-Cox *et al.*, 2007; Taipale *et al.*, 2021; Rahman *et al.*, 2022), of which three (Wang *et al.*, 2002; Hippisley-Cox *et al.*, 2007; Rahman *et al.*, 2022) had adjusted for obesity – suggested to be associated with an increased risk of breast cancer (Iyengar *et al.*, 2019), whilst substance misuse or smoking have been adjusted in four studies (Mortensen, 1987; Hippisley-Cox *et al.*, 2007; Taipale *et al.*, 2021; Rahman *et al.*, 2022). Of the five studies with adjustment for lifestyle factors, four studies reported a significant association between antipsychotic use and breast cancer risk.

Socioeconomic factors were mostly represented by occupation, income, education status or a summarised Townsend score. Six studies (Mortensen, 1987; Wang *et al.*, 2002; Hippisley-Cox *et al.*, 2007; Chou *et al.*, 2017; Pottegård *et al.*, 2018; Rahman *et al.*, 2022) adjusted for socioeconomic status, of which five (Wang *et al.*, 2002; Hippisley-Cox *et al.*, 2007; Chou *et al.*, 2017; Pottegård *et al.*, 2018; Rahman *et al.*, 2022) reported a significant association between antipsychotic use and breast cancer risk.

### Exposure – antipsychotic use

Antipsychotic use was defined with electronic records in eight out of the nine studies (Mortensen, 1987; Wang *et al.*, 2002; Dalton *et al.*, 2006; Hippisley-Cox *et al.*, 2007; Chou *et al.*, 2017; Pottegård *et al.*, 2018; Taipale *et al.*, 2021; Rahman *et al.*, 2022), the remaining study (George *et al.*, 2020) used self-reported antipsychotic use to determine the exposure group. All studies took any antipsychotic use into account. Exposure durations were specified in three studies, Wang *et al*. included participants with at least 3 months' exposure to antipsychotics prior to the index date from which the follow-up started (Wang *et al.*, 2002); Dalton *et al.* only included participants who had received at least two prescriptions (Dalton *et al.*, 2006); and Taipale *et al*. considered participants with prior antipsychotic exposure until 1 year before breast cancer diagnosis, with a case control design (Taipale *et al.*, 2021).

The following variables were used to represent the extent of exposure for further stratification of the exposed group: cumulative doses (Wang *et al.*, 2002; Pottegård *et al.*, 2018), average yearly dosage (Mortensen, 1987; Chou *et al.*, 2017), prescription count (Dalton *et al.*, 2006), duration (Taipale *et al.*, 2021) and prolactin-elevating propensity (Rahman *et al.*, 2022). Two remaining studies (Hippisley-Cox *et al.*, 2007; George *et al.*, 2020) included participants with any use of antipsychotics without further stratifying by the extent of exposure in their exposed groups. Of the two studies that did not stratify participants by the extent of exposure, one reported a significant association (OR 1.55, 95% CI 1.08–2.23) (Hippisley-Cox *et al.*, 2007). Five out of the seven studies (Wang *et al.*, 2002; Chou *et al.*, 2017; Pottegård *et al.*, 2018; Taipale *et al.*, 2021; Rahman *et al.*, 2022) that stratified participants by the extent of exposure reported significant associations of antipsychotic use with breast cancer.

Despite a null association with the exposure defined as any antipsychotic use, long-term use (defined as having a cumulative dose of over 10 000 mg of olanzapine equivalents) was found to have a small association with breast cancer development in Pottegård *et al.* (2018). An increased risk with prolonged exposure was also suggested in two other studies (Wang *et al.*, 2002; Taipale *et al.*, 2021). Taipale *et al.* reported ORs 1.18 (95% CI 0.86–1.62) for 1–4 years of antipsychotic use and 1.74 (95% CI 1.38–2.21) for at least 5 years of antipsychotic use (Taipale *et al.*, 2021), and Wang *et al*. showed an increased risk with at least 6 years of antipsychotic exposure (HR 2.37, 95% CI 1.25–4.47), whereas breast cancer risk amongst antipsychotic users of less than 6 years were reported to be non-significant. In contrast, the dose–response relationship was not observed in the atypical antipsychotic subgroup of Chou *et al*., where an apparent association was observed with lower exposure instead of increased exposure. They reported HRs 2.49 (95% CI 1.69–3.66) and 1.05 (95% CI 0.58–1.87) for mean antipsychotic exposure of less than 28 and greater than 245 g/year, respectively.

Some studies have also investigated the prolactin-elevating properties of antipsychotics and its association with breast cancer development (Chou *et al.*, 2017; Pottegård *et al.*, 2018; Taipale *et al.*, 2021; Rahman *et al.*, 2022). Exposure to antipsychotics with prolactin-elevating properties were included in Pottegård *et al.* (2018), to which long-term exposure showed an increased risk of breast cancer. Rahman *et al*. grouped exposure according to prolactin-elevating propensity into three categories of low, medium and high propensity. They reported that users of antipsychotics with medium and high prolactin-elevating properties were significantly associated with breast cancer development (Rahman *et al.*, 2022). Taipale *et al.* (2021) compared prolonged periods of prolactin-increasing antipsychotic use to those exposed for less than a year. The results showed an increased risk amongst those exposed for at least 5 years (OR 1.56, 95% CI 1.27–1.92), corresponding to the results seen in Pottegård *et al*. Prolactin-elevating antipsychotics reported in Chou *et al.* were defined as risperidone, paliperidone or amisulpride, the study compared schizophrenia patients exposed to said antipsychotics to a non-schizophrenia cohort as the non-exposed comparator, the results indicate a significant association in the use of the three prolactin-elevating antipsychotics with breast cancer development (HR 1.96, 95% CI 1.36–2.82) (Chou *et al.*, 2017).

### Quality assessment scores

All nine studies received a satisfactory quality assessment score of seven to nine stars (Wang *et al.*, 2002; Dalton *et al.*, 2006; Hippisley-Cox *et al.*, 2007; Chou *et al.*, 2017; Pottegård *et al.*, 2018; George *et al.*, 2020; Taipale *et al.*, 2021; Rahman *et al.*, 2022). One case-control study (Mortensen, 1987) received a lower score of two out of four stars in regards to the selection of cases and controls and the limited representativeness of the cases due to its small sample size. All studies had adjusted for both age and other covariates associated with the risk of breast cancer such as comorbidity or concurrent medication.

### Pooled estimates of the association

**Fig. 2.**
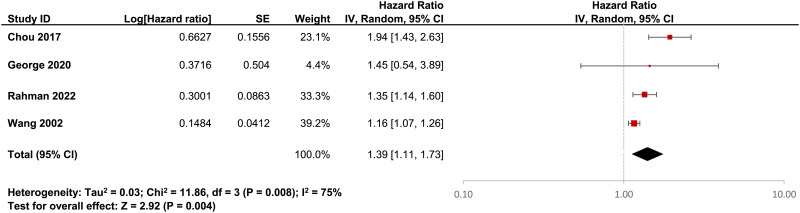
Forest plot showing HRs generated from retrieved individual cohort studies (*n* = 4) using Cox proportional hazard models and the pooled HR. For George *et al*. (2020), the HR for atypical antipsychotic use and invasive breast cancer was used.

**Fig. 3.**
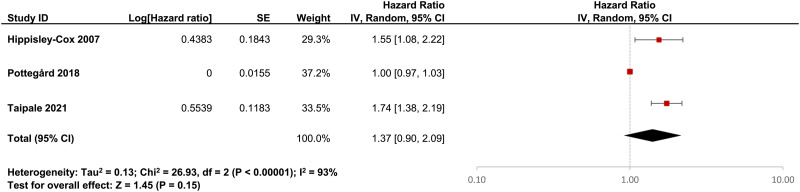
Forest plot showing ORs generated from retrieved individual case-control studies (*n* = 3) using logistic regression and the pooled OR.

Using a random effects model, we pooled the HRs and ORs of breast cancer between antipsychotic users and non-users from four cohort studies (Wang *et al.*, 2002; Chou *et al.*, 2017; George *et al.*, 2020; Rahman *et al.*, 2022) and three case-control studies (Hippisley-Cox *et al.*, 2007; Pottegård *et al.*, 2018; Taipale *et al.*, 2021) respectively, with the *I*^2^ estimated at 75 and 93%. Figures 2 and 3 show the forest plots for the pooled estimate as well as the estimated ratios reported by individual studies. Results suggest a moderate association of antipsychotic use (*v*. non-use) with breast cancer with a >30% increased risk observed, although the pooled OR did not reach statistical significance (HR 1.39, 95% CI 1.11–1.73; OR 1.37, 95% CI 0.90–2.09). As only three and four studies were included in the pooled estimates of the OR and HR, we did not conduct the Egger's regression test for publication bias.

As one of the cohort studies (George *et al.*, 2020) stratified the analysis by typical/atypical antipsychotics and invasive/in-situ breast cancer, we included the HR for atypical antipsychotics and invasive breast cancer in that study for the pooled estimate and replicated the analysis with all three other HRs separately as a sensitivity analysis to test for the robustness of the results. No substantial difference was observed as shown in online Supplementary eTable 2.

## Discussion

Results of this review support the association between the use of antipsychotic medications and an increased risk of breast cancer. Six out of nine included studies of a good quality reported a significant association. Evidence shows a further extent of exposure to antipsychotics, such as a longer duration of use, is associated with a higher risk of breast cancer, particularly for antipsychotics with prolactin-elevating properties. Outcome definition, exposure operationalisation and quality assessment score did not have a noticeable effect on the difference in results between the studies. From the meta-analysis, we estimated a moderate positive association of antipsychotic use and breast cancer with a >30% elevated risk.

Antipsychotics are dopamine receptor antagonists and prohibit the binding of dopamine to dopamine D2 receptors (D2R), this action increases prolactin secretion (Besnard *et al.*, 2013). Typical antipsychotics were reported to have higher occurrences of elevated serum prolactin levels (hyperprolactinaemia) in comparison with atypical antipsychotic users (Madhusoodanan *et al.*, 2010; Manu, 2012; Vuk Pisk *et al.*, 2019; Dehelean *et al.*, 2020). Compared with typical antipsychotics, the majority of atypical antipsychotics present fewer prolactin related side effects, hypothesised to be due to a shorter binding duration between the drug and D2R (Bargiota *et al.*, 2013). Atypical antipsychotics have a higher risk of inducing metabolic syndrome, including central obesity and hyperlipidaemia, than typical antipsychotics (De Hert *et al.*, 2012; Wei Xin Chong *et al.*, 2016), both of which have been investigated to have a potentially increased risk of breast cancer (Iyengar *et al.*, 2019; Chowdhury *et al.*, 2021). Moreover, studies on schizophrenia patients showed that the risk of developing cardiovascular disease as well as type-2 diabetes mellitus of individuals was higher in atypical antipsychotic drugs (Drici and Priori, 2007; De Hert *et al.*, 2012), with recent literature suggesting an association between diabetes and breast cancer risk (Liao *et al.*, 2011). Hence, the association between antipsychotic use and breast cancer may possibly be explained by more than one physiological mechanism. With a majority of the included studies in this review having made reasonable adjustments for potential confounders such as clinical history, lifestyle factors and socioeconomic background, with several studies reporting increased breast cancer risk in prolactin-elevating antipsychotics (Pottegård *et al.*, 2018; Taipale *et al.*, 2021; Rahman *et al.*, 2022), the observed association may likely be attributed to these biological mechanisms as described.

With an increasingly prevalent use of antipsychotic medications worldwide, the risk of adverse events associated with it should be investigated in more breadth and depth to inform clinical practice. This study on the potentially elevated risk of breast cancer adds to the current knowledge of adverse events associated with antipsychotic use, such as stroke and myocardial infarction were investigated previously (Douglas and Smeeth, 2008; Sørensen *et al.*, 2013; Lai *et al.*, 2020), and use of prolactin-inducing antipsychotics was also reported to be associated with hip fractures (De Hert *et al.*, 2016*a*). Given the potentially multifold underlying physiological mechanisms underlying the side effects, a comprehensive holistic assessment of the clinical profile of the patients should be made along with the safety profile of specific antipsychotics to optimise the treatment outcome (Huhn *et al.*, 2019). Interestingly, the elevated breast cancer risk observed in this study may not be applicable to other cancer types. In fact, a lower risk of lung and other cancers have been found associated with the use of antipsychotics and there are ongoing efforts in drug repurposing to experiment the cancer prevention properties of antipsychotic medications (Li *et al.*, 2022). The exact mechanism of this inverse relationship is largely unclear.

The increased use of routine electronic health records in pharmacovigilance studies have contributed to the existing literature significantly, as shown in the included studies in this review. While providing a typically large sample size with realistic real-world clinical data, there are intrinsic limitations to these records. Specifically, the lack of lifestyle and other important factors might introduce bias to the estimated association. Primary data collection may provide much more detailed information but with a much-limited sample size. Therefore, both types of research are much warranted, and the evidence needs to be considered in the context of a variety of study designs with various strengths and weaknesses for a balanced overall assessment. With the benefits of record-linkage techniques with prescription registries, antipsychotic prescription practices such as antipsychotic polypharmacy in comparison with monotherapy can be addressed in future studies. One review suggested that aripiprazole use in combination with another antipsychotic was associated with better lipid profile outcomes than the use of other antipsychotic polypharmacy or monotherapy, although the quality of evidence was lacking (Ijaz *et al.*, 2018). Further investigation in this area could possibly provide a more substantiated association.

### Limitations

In spite of the important clinical implications, there are several limitations. First, the reviewed evidence is all generated from observational research without randomisation. There is likely unmeasured confounding effects and causal inferences need to be made with great caution. Specifically, the comparators selected for some included studies may not be entirely suitable and could be subject to potential selection bias. One example of mitigating this bias is demonstrated in Rahman *et al*. through the use of anticonvulsants and lithium as comparator drugs, which are also prescribed to patients with psychiatric disorders such as anxiety, depression and bipolar disorder, but with no known risk of hyperprolactinaemia (Ajmal *et al.*, 2014). Second, the rare incidence of male breast cancer cases, even in very large electronic health record databases, poses as a challenge to derive a meaningful statistical analysis. Despite having included studies with male breast cancer cases in this review, the association of antipsychotic use with breast cancer amongst the male population would be difficult to conclude.

There are also limitations specific to this review as well. First, although the meta-analysis generated consistent results across study designs, i.e. cohort and case-control, the association could not be appropriately pooled across designs to increase the precision of the estimate. Second, the number of studies is too small to provide a more precise estimate of the hypothesised association and the presence of publication bias could not be tested as a result. Third, significant heterogeneity was observed between studies even within the same design, probably due to different populations, research practice and availability of data, further studies with more accrued data should investigate factors that contribute to this heterogeneity. Recent studies reported higher basal epigenetic changes in African American women (Joshi *et al.*, 2022), a population found to have the highest rates of BRCA genetic mutations (Fackenthal and Olopade, 2007), which could increase the risk of breast cancer development. Varying degrees of risk in certain breast cancer subtypes between women of Hispanic, Asian, Black and White descent were also reported (Kurian *et al.*, 2010). The variation in breast cancer risk between ethnicities is suggestive of biological heterogeneities; further exploration may be warranted for clarification on the potential differences with regards to the observed association. Fourth, we only examined studies written in English language. Further reviews including other languages may be warranted.

## Conclusion

In conclusion, we found a moderate association between the use of antipsychotics and breast cancer with a more evident association observed with prolactin-elevating medications and greater extent of antipsychotic exposure. This risk, together with other known associated adverse events, should be weighed against the anticipated treatment outcomes for a balanced clinical management decision.

## Data Availability

All data used in the systematic review and meta-analyses can be found in the included studies.

## Supplementary material

For supplementary material accompanying this paper visit https://doi.org/10.1017/S2045796022000476. click here to view supplementary material
