# Association of antipsychotic use with breast cancer: a systematic review and meta-analysis of observational studies with over 2 million individuals—CORRIGENDUM

**DOI:** 10.1017/S2045796022000531

**Published:** 2022-09-27

**Authors:** Janice Ching Nam Leung, Dora Wai Yee Ng, Rachel Yui Ki Chu, Edward Wai Wa Chan, Lei Huang, Dawn Hei Lum, Esther Wai Yin Chan, Daniel J. Smith, Ian Chi Kei Wong, Francisco Tsz Tsun Lai

**Affiliations:** 1 Centre for Safe Medication Practice and Research, Department of Pharmacology and Pharmacy, Li Ka Shing Faculty of Medicine, The University of Hong Kong, Hong Kong SAR, People's Republic of China; 2 Laboratory of Data Discovery for Health (D24H), Hong Kong Science Park, Hong Kong Science and Technology Park, Hong Kong SAR, People's Republic of China; 3 Centre for Clinical Brain Sciences, Division of Psychiatry, College of Medicine & Veterinary Medicine, The University of Edinburgh, Edinburgh, Scotland, UK; 4 Research Department of Practice and Policy, School of Pharmacy, University College London, London, UK; 5 Aston School of Pharmacy, Aston University, Birmingham, UK

There is a typo in one of the headings in Figure 3 on p. 9. The correct last heading is not ‘Hazard Ratio’ but ‘Odds Ratio’. The authors apologize for the mistake.

## References

[ref1] Leung, J., Ng, D., Chu, R., Chan, E., Huang, L., Lum, D., . . . Lai, F. (2022) Association of antipsychotic use with breast cancer: A systematic review and meta-analysis of observational studies with over 2 million individuals. Epidemiology and Psychiatric Sciences, 31, E61. doi:10.1017/S2045796022000476 36059215 PMC9483823

