# Supplementary material for: Association of antipsychotic use with breast cancer: a systematic review and meta-analysis of observational studies with over 2 million individuals
Source: Epidemiol Psychiatr Sci. 2022 Sep 5;31:e61. doi: 10.1017/S2045796022000476 (PMC9483823; doi:10.1017/S2045796022000476)
Supplement: Supplementary file 1 [file S2045796022000476sup001.docx]

## SUPPLEMENTAL TABLES

| **eTable 1**. Electronic database search keywords | |
| --- | --- |
| Databases | Keywords |
| PubMed | 1. All field: antipsychotic OR neuroleptic OR tranquilizer OR "tranquillizing agent" OR psychotropic OR "dopamine receptor blocker" OR "dopamine antagonist" OR "dopamine receptor blocking agent" OR "D2 receptor blocker" OR "D2 antagonist" OR "D2 receptor blocking agent" OR "typical antipsychotic" OR "atypical antipsychotic" OR "first generation antipsychotic" OR "second generation antipsychotic" OR benperidol OR clopenthixol OR flupentixol OR fluspiriline OR methotrimeprazine OR perazine OR sertindole OR zotepine OR chlorpromazine OR droperidol OR fluphenazine OR haloperidol OR loxapine OR perphenazine OR pimozide OR prochlorperazine OR thiothixene OR tiotixene OR thioridazine OR trifluoperazine OR molindone OR levomepromazine OR zuclopenthixol OR apiprazole OR asenapine OR clozapine OR iloperidone OR olanzapine OR paliperidone OR quetiapine OR risperidone OR ziprasidone OR amisulpride OR brexpiprazole OR cariprazine OR lumateperone OR pimavanserin OR lurasidone OR Thorazine OR Largactil OR Inapsine OR Modecate OR Moditen OR Prolixin OR RhoFluphenazine OR Haldol OR Serenace OR Loxitane OR Adasuve OR Trilafon OR Orap OR Stemetil OR Buccastem OR Navane OR Mellaril OR Melleril OR Stelazine OR Moban OR Neurocil OR Cisordinol OR Clopixol OR Abilify OR Saphris OR Clozaril OR "FazaClo ODT" OR Versacloz OR Fanapt OR Zomaril OR Zyprexa OR Invega OR Seroquel OR Risperdal OR Geodon OR Solian OR Barhemsys OR Rexulti OR Vraylar OR Caplyta OR Nuplazid OR Latuda OR Flupenthixol OR Depixol OR Fluanxol OR Nozinan OR Levoprome OR Detenler OR Hirnamin OR Levotomin OR Dogmatil OR Serdolect OR Zoleptil 2. MeSH: "Antipsychotic Agents"[MeSH] OR "Tranquilizing Agents"[MeSH] OR "Phenothiazines"[MeSH] OR "Penfluridol"[MeSH] OR "Molindone"[MeSH] OR "Butyrophenones"[MeSH] 3. 1 OR 2 4. Title/abstract: "cancer risk" OR "cancer incidence" OR cancer OR tumor OR tumour OR neoplasm OR carcinoma OR malignancy OR tumorigenesis OR carcinogenesis 5. All field: "breast cancer" OR "breast tumor" OR "breast tumour" OR "breast lesion" OR "breast neoplasm" OR "breast carcinoma" OR "breast malignancy" OR "mammary cancer" OR "mammary tumor" OR "mammary tumour" OR "mammary lesion" OR "mammary neoplasm" OR "mammary carcinoma" OR "mammary malignancy" OR "ductal carcinoma" OR "lobular carcinoma" OR "breast cancer risk" OR "breast cancer incidence" OR "mammogra*" OR "breast tumorigenesis" OR "breast carcinogenesis" OR "cancer of breast" OR "cancer of the breast" OR "malignant tumor of breast" OR "malignant tumour of breast" 6. 4 AND 5 7. MeSH: "Breast Neoplasms"[MeSH] OR "Breast Cancer Lymphedema"[MeSH] OR "Unilateral Breast Neoplasms"[MeSH] OR "Triple Negative Breast Neoplasms"[MeSH] OR "Inflammatory Breast Neoplasms"[MeSH] OR "BRCA1 Protein"[MeSH] OR "BRCA2 Protein"[MeSH] OR "Breast Neoplasms, Male"[MeSH] 8. 6 OR 7 9. 3 and 8 |
| EMBASE | 1. "neuroleptic agent"(explode) 2. antipsychotic OR neuroleptic OR tranquilizer OR "tranquillizing agent" OR psychotropic OR "dopamine receptor blocker" OR "dopamine antagonist" OR "dopamine receptor blocking agent" OR "D2 receptor blocker" OR "D2 antagonist" OR "D2 receptor blocking agent" OR "typical antipsychotic" OR "atypical antipsychotic" OR "first generation antipsychotic" OR "second generation antipsychotic" OR benperidol OR clopenthixol OR flupentixol OR fluspiriline OR methotrimeprazine OR perazine OR sertindole OR zotepine OR chlorpromazine OR droperidol OR fluphenazine OR haloperidol OR loxapine OR perphenazine OR pimozide OR prochlorperazine OR thiothixene OR tiotixene OR thioridazine OR trifluoperazine OR molindone OR levomepromazine OR zuclopenthixol OR apiprazole OR asenapine OR clozapine OR iloperidone OR olanzapine OR paliperidone OR quetiapine OR risperidone OR ziprasidone OR amisulpride OR brexpiprazole OR cariprazine OR lumateperone OR pimavanserin OR lurasidone OR Thorazine OR Largactil OR Inapsine OR Modecate OR Moditen OR Prolixin OR RhoFluphenazine OR Haldol OR Serenace OR Loxitane OR Adasuve OR Trilafon OR Orap OR Stemetil OR Buccastem OR Navane OR Mellaril OR Melleril OR Stelazine OR Moban OR Neurocil OR Cisordinol OR Clopixol OR Abilify OR Saphris OR Clozaril OR "FazaClo ODT" OR Versacloz OR Fanapt OR Zomaril OR Zyprexa OR Invega OR Seroquel OR Risperdal OR Geodon OR Solian OR Barhemsys OR Rexulti OR Vraylar OR Caplyta OR Nuplazid OR Latuda OR Flupenthixol OR Depixol OR Fluanxol OR Nozinan OR Levoprome OR Detenler OR Hirnamin OR Levotomin OR Dogmatil OR Serdolect OR Zoleptil (mp) 3. 1 OR 2 4. "breast cancer"(explode) 5. "cancer risk"(explode) 6. "cancer incidence" (explode) 7. 5 OR 6 8. 4 AND 7 9. 3 AND 8 |
| Web of Science ™ | 1. Topic: antipsychotic OR neuroleptic OR tranquilizer OR "tranquillizing agent" OR psychotropic OR "dopamine receptor blocker" OR "dopamine antagonist" OR "dopamine receptor blocking agent" OR "D2 receptor blocker" OR "D2 antagonist" OR "D2 receptor blocking agent" OR "typical antipsychotic" OR "atypical antipsychotic" OR "first generation antipsychotic" OR "second generation antipsychotic" OR benperidol OR clopenthixol OR flupentixol OR fluspiriline OR methotrimeprazine OR perazine OR sertindole OR zotepine OR chlorpromazine OR droperidol OR fluphenazine OR haloperidol OR loxapine OR perphenazine OR pimozide OR prochlorperazine OR thiothixene OR tiotixene OR thioridazine OR trifluoperazine OR molindone OR levomepromazine OR zuclopenthixol OR apiprazole OR asenapine OR clozapine OR iloperidone OR olanzapine OR paliperidone OR quetiapine OR risperidone OR ziprasidone OR amisulpride OR brexpiprazole OR cariprazine OR lumateperone OR pimavanserin OR lurasidone OR Thorazine OR Largactil OR Inapsine OR Modecate OR Moditen OR Prolixin OR RhoFluphenazine OR Haldol OR Serenace OR Loxitane OR Adasuve OR Trilafon OR Orap OR Stemetil OR Buccastem OR Navane OR Mellaril OR Melleril OR Stelazine OR Moban OR Neurocil OR Cisordinol OR Clopixol OR Abilify OR Saphris OR Clozaril OR "FazaClo ODT" OR Versacloz OR Fanapt OR Zomaril OR Zyprexa OR Invega OR Seroquel OR Risperdal OR Geodon OR Solian OR Barhemsys OR Rexulti OR Vraylar OR Caplyta OR Nuplazid OR Latuda OR Flupenthixol OR Depixol OR Fluanxol OR Nozinan OR Levoprome OR Detenler OR Hirnamin OR Levotomin OR Dogmatil OR Serdolect OR Zoleptil 2. Topic: "cancer risk" OR "cancer incidence" OR cancer OR tumor OR tumour OR neoplasm OR carcinoma OR malignancy OR tumorigenesis OR carcinogenesis 3. All field: "breast cancer" OR "breast tumor" OR "breast tumour" OR "breast lesion" OR "breast neoplasm" OR "breast carcinoma" OR "breast malignancy" OR "mammary cancer" OR "mammary tumor" OR "mammary tumour" OR "mammary lesion" OR "mammary neoplasm" OR "mammary carcinoma" OR "mammary malignancy" OR "ductal carcinoma" OR "lobular carcinoma" OR "breast cancer risk" OR "breast cancer incidence" OR "mammogra*" OR "breast tumorigenesis" OR "breast carcinogenesis" OR "cancer of breast" OR "cancer of the breast" OR "malignant tumor of breast" OR "malignant tumour of breast" 4. 1 AND 2 AND 3 |

| **eTable 2**. Sensitivity analysis for the pooled hazard ratio (HR) with alternative HRs ^a^ provided by George et al. 2020 being adopted in meta-analysis | | |
| --- | --- | --- |
|  | HR in George et al. 2020 | Resulted pooled HR |
| Typical antipsychotics with invasive breast cancer | 0.67 (0.36, 1.25) | 1.22 (1.13, 1.30) |
| Typical antipsychotics with in-situ breast cancer | 2.05 (0.97, 4.30) | 1.23 (1.15, 1.32) |
| ^a^ Atypical antipsychotics with in-situ breast cancer unavailable from study | | |

| **eTable 3**. Details of the quality assessment procedures using the Newcastle Ottawa Scale | | |
| --- | --- | --- |
| Section | Case-control studies | Cohort studies |
| Selection | - - - 1. Case definition: Allocate one mark if cases are defined with a record of diagnosis (e.g.: diagnostic code) or with independent validation | Representativeness of exposed cohort:  Allocate one mark if sample size of exposed participants > 500 |
|  | - - - 1. Representativeness of cases: Allocate one mark if sample size > 100 | 1. Selection of the non-exposed cohort: Allocate one mark if non-exposed participants were drawn from the same community as the exposed cohort |
|  | 1. Selection of controls:   Allocate one mark if community controls were used, no marks given if hospital controls were used/no description provided | - - - 1. Ascertainment of exposure:   Allocate one mark if study determines exposure via secure records (e.g.: hospital pharmacy records/record linkage to prescription registry) |
|  | 1. Definition of controls:   Allocate one mark if controls had no history of breast cancer | 1. Demonstration that outcome of interest was not present at start of study:   Allocate one mark if all participants had no history of breast cancer at study index date |
| Comparability | 1. Study controls for important factors:   Allocate one mark if study controls for age, comorbidities, and concurrent medications | 1. Study controls for important factors:   Allocate one mark if study controls for age, comorbidities, and concurrent medications |
|  | 1. Study controls for additional factors:   Allocate one mark if study controls for factors other than those listed as important factors | 1. Study controls for additional factors:   Allocate one mark if study controls for factors other than those listed as important factors |
| Exposure (Case-control)/ Outcome (Cohort) | 1. Ascertainment of exposure:   Allocate one mark if study determines exposure via secure records (e.g.: pharmacy records/data-linkage to prescription registry) | 1. Assessment of outcome:   Allocate one mark if outcome is defined with a record of diagnosis (e.g.: diagnostic code) or with independent validation |
|  | 1. Same method of ascertainment for cases and controls:   Allocate one mark if the same method of ascertainment for cases and controls’ exposure were used | 1. Was follow-up long enough for outcomes to occur:   Allocate one mark if follow-up time > 1 year |
|  | 1. Non-Response rate:   Allocate one mark if non-response rate was specified | 1. Adequacy of follow up of cohorts:   Allocate one mark if complete follow up; or < 20% of participants were lost to follow up is reported |
|  | | |
